# Timelike Thin-Shell Evolution in Gravitational Collapse: Classical Dynamics and Thermodynamic Interpretation

**DOI:** 10.3390/e28010096

**Published:** 2026-01-13

**Authors:** Axel G. Schubert

**Affiliations:** Independent Researcher, 40589 Düsseldorf, Germany; schubert.ag@posteo.de

**Keywords:** timelike thin shell, junction geometry, static patch, classical collapse endpoint, Schwarzschild–de Sitter, curvature invariants, negative heat capacity, quasi-trapped modes

## Abstract

This work explores late-time gravitational collapse using timelike thin-shell methods in classical general relativity. A junction surface separates a regular de Sitter interior from a Schwarzschild or Schwarzschild–de Sitter exterior in a post-transient regime with fixed exterior mass *M* (ADM for $\Lambda_{+}=0$), modelling a vacuum–energy core surrounded by an asymptotically classical spacetime. The configuration admits a natural thermodynamic interpretation based on a geometric area functional $S_{\mathrm{shell}}\propto R^{2}$ and Tolman redshift, both derived from classical junction conditions and used as an entropy-like coarse-grained quantity rather than a fundamental statistical entropy. Key results include (i) identification of a deceleration mechanism at the balance radius $R_{\mathrm{thr}}={\left(3M/\Lambda_{-}\right)}^{1/3}$ for linear surface equations of state p=wσ; (ii) classification of the allowable radial domain V(R)≤0 for outward evolution; (iii) bounded curvature invariants throughout the shell-supported spacetime region; and (iv) a mass-scaled frequency bound $f_{c}R_{S}\leq \xi /\left(3\sqrt{3}\;\pi \right)$ for persistent near-shell spectral modes. All predictions follow from standard Israel junction techniques and provide concrete observational tests. The framework offers an analytically tractable example of regular thin-shell collapse dynamics within classical general relativity, with implications for alternative compact object scenarios.

## 1. Introduction

Thermodynamic principles have long influenced the understanding of gravitational systems, especially through insights gained from black hole physics. Concepts such as entropy, temperature, and energy flux have revealed a deep and sometimes unexpected connection between gravity and thermodynamics [1,2,3]. Seminal results—such as the proportionality between horizon area and entropy or the appearance of Hawking radiation—have strengthened the perspective that spacetime itself may admit a thermodynamic description, at least in certain regimes.

Even outside quantum contexts, purely classical self-gravitating systems exhibit thermodynamic features. For instance, they can display negative specific heat, gravitational redshift, and entropy maxima under constraints [4,5]. These properties suggest that certain aspects of gravitational thermodynamics may emerge from general relativity alone, without recourse to quantum effects.

The connection between gravitational dynamics and entropy carries profound implications for understanding collapse endpoints and thermodynamic behavior in curved spacetimes. While classical general relativity cannot access the microscopic details underlying quantum–mechanical entropy, coarse-graining physical quantities—such as the shell’s trajectory and stress–energy fluctuations—provides a “macroscopic” thermodynamic description that parallels the area law established for black holes [6] and related proposals for gravitational entropy [7]. It is also consistent with the area scaling of the entanglement entropy of quantum fields across spatial regions [8]. This perspective suggests that gravitational entropy can be studied and quantified within purely classical setups, offering a foundational bridge to quantum refinements as proposed in holographic or statistical approaches.

This study investigates whether classical general relativity, in a highly symmetric and controlled setting, supports such thermodynamic behavior during late-stage gravitational collapse. Specifically, the focus is placed on a spherically symmetric timelike thin shell, governed by Israel’s junction conditions [9,10], which separates a regular de Sitter interior from an exterior Schwarzschild or Schwarzschild–de Sitter region. Within the shell-supported static domain, this setup replaces the central Schwarzschild singularity by a constant-curvature core and allows for a fully analytic treatment of the system’s evolution. From a physical point of view, the de Sitter interior can be interpreted as an effective high-density vacuum–energy core with equation of state p≃−ρ, while the timelike thin shell idealizes a narrow transition layer between this core and the exterior vacuum geometry, in the spirit of related de Sitter–core models [11].

Alternative approaches to gravitational collapse endpoints have been extensively studied, including gravastars with exotic equations of state [11], boson stars and other self-gravitating solitons [12], and quantum-corrected scenarios invoking modified gravity or stringy effects [13,14]. While these models address important conceptual challenges, they often rely on additional assumptions beyond classical general relativity, require numerical input with model-dependent choices, or do not lead to sharp, falsifiable signatures. Here the aim is deliberately narrower: this work asks what can already be derived in a fully classical, junction-controlled setting once a regular de Sitter core is matched to an asymptotically classical exterior and the shell remains timelike in the static patches. In this regime the system is analytically tractable and yields concrete observational consequences.

The thermodynamic-like interpretation developed below is likewise macroscopic and classical. It follows from the area scaling of a geometric entropy-like functional and Tolman redshift, providing a controlled coarse-graining of the shell evolution without appealing to quantum microstates or exotic physics.

After an initial transient formation period, the exterior mass is treated as fixed, modelling a phase of negligible net inflow. The subsequent evolution is then captured by an effective-potential description, from which one can derive conditions for outward motion, thermodynamic scaling relations, and an observable frequency cutoff. All results are obtained within classical general relativity using standard junction techniques.

Concretely, the analysis yields the following:

- A deceleration mechanism at a characteristic balance radius set by the exterior mass and the interior cosmological constant, providing analytical insight into the late-time shell dynamics.
- An analytic characterization of the admissible radial domain for evolution, determined by the effective potential and the static-patch restrictions.
- Bounded scalar curvature invariants across the shell-supported static region considered here, under the stated assumptions on the evolution domain and surface stresses.
- A falsifiable observational consequence: persistent near-shell spectral features observed at infinity must satisfy a specific mass-scaled upper bound, providing a concrete test.

The model naturally supports a thermodynamic interpretation through entropy–area scaling, Tolman redshift effects, and effective transport properties, while remaining within classical general relativity. Together with the analytic results summarized above, this offers a controlled framework for examining gravitational thermodynamics, information-theoretic aspects of collapse scenarios, and classical limits of entropy production through coarse-graining mechanisms.

## 2. Geometric Setup and Methods

We consider a spherically symmetric thin-shell spacetime constructed by matching a regular de Sitter interior to an exterior Schwarzschild or Schwarzschild–de Sitter geometry. The spacetime is divided into three regions: an interior $\left(M_{-}\right)$ with cosmological constant $\Lambda_{-}$ and vanishing mass, a timelike thin shell at radius R(τ) with proper time τ, and an exterior $\left(M_{+}\right)$ characterized by total mass *M* and cosmological constant $\Lambda_{+}$. The metric in each region takes the static form

(1) $$ds_{\pm }^{2}=-f_{\pm }\left(r\right)\;dt_{\pm }^{2}+\frac{dr^{2}}{f_{\pm }\left(r\right)}+r^{2}\;d\Omega^{2},$$

where

(2) $$f_{\pm }\left(r\right)=1-\frac{2M_{\pm }}{r}-\frac{\Lambda_{\pm }r^{2}}{3}.$$

We fix $M_{-}=0$ in the interior and $M_{+}=M$ in the exterior. The shell is assumed to be infinitesimally thin and characterized by surface energy density σ(τ) and pressure p(τ). Throughout we restrict attention to the static domain $f_{\pm }\left(R\right)>0$, so that the shell remains timelike and outside any exterior horizons.

### 2.1. Junction Conditions and Shell Dynamics

The motion of the shell is governed by the Israel junction conditions [9,15,16], which relate the jump in extrinsic curvature across the shell to its surface stress–energy tensor. With outward-pointing unit normals chosen so that they point from the de Sitter interior to the Schwarzschild/Schwarzschild–de Sitter exterior, the junction condition can be written as

$$\sqrt{f_{+}\left(R\right)+{\dot{R}}^{2}}-\sqrt{f_{-}\left(R\right)+{\dot{R}}^{2}}=\kappa \left(R\right),$$

where

(3) κ(R):=4πσ(R)R.

Here we explicitly take the positive square-root branch for both $\sqrt{f_{\pm }\left(R\right)+{\dot{R}}^{2}}$ and the outward-pointing unit normal, corresponding to the standard, outward-embedded shell with positive surface energy density and a non-exotic sign for the jump in extrinsic curvature. The opposite sign choices would describe inward-embedded configurations or require exotic matter with negative effective energy density, which are not considered here.

Solving the junction condition for ${\dot{R}}^{2}$ yields an energy-balance equation of the form

(4) $${\dot{R}}^{2}+V\left(R\right)=0,$$

where V(R) is the effective potential determined by the geometry and shell content. Following standard thin-shell techniques [15], it takes the form

(5) $$V\left(R\right)=f_{-}\left(R\right)-\frac{{\left(f_{+}\left(R\right)-f_{-}\left(R\right)-\kappa {\left(R\right)}^{2}\right)}^{2}}{4\kappa {\left(R\right)}^{2}}.$$

In addition to the normal-jump condition, the shell stress–energy must satisfy intrinsic energy–momentum conservation on the worldvolume,

(6) DaS  ba=−Tμνe  bμnν−+,

where $D_{a}$ is the covariant derivative compatible with the induced metric on the shell, e  bμ are tangential basis vectors, $n^{\mu }$ is the unit normal, and ${\left[\;\cdot \;\right]}_{-}^{+}$ denotes the jump across the shell [9,15,16]. In spherical symmetry this identity reduces to a surface energy–balance (first-law–type) relation

(7) $$\frac{d(\sigma A)}{d\tau }+p\;\frac{dA}{d\tau }=\Phi \;A,\;A=4\pi R^{2},$$

where Φ is the net normal energy-flux density across the shell measured in the shell rest frame. Equivalently, Φ may be written as the jump of the mixed normal–tangential projection of the bulk stress tensor,

(8) $$\Phi \;:=\;-{\left[T_{\mu \nu }u^{\mu }n^{\nu }\right]}_{-}^{+},$$

with $u^{\mu }$ the shell four-velocity (i.e. uμ=e  τμ) and $n^{\mu }$ the outward unit normal. With this convention, Φ>0 corresponds to net inflow into the shell, while Φ<0 corresponds to net outflow. In the post-transient regime considered in most of the dynamical analysis below, we assume negligible net inflow so that Φ≃0; the explicit flux term is retained to keep the first-law form manifest and to discuss robustness under weak inflow (Section 7).

In the case of a linear surface equation of state p=wσ and negligible net flux (Φ≃0), the surface energy balance (7) implies

(9) $$\sigma \left(R\right)\propto R^{-2(1+w)},$$

so that κ(R) scales as $R^{-(1+2w)}$ up to a constant prefactor. In the dynamical analysis below we will restrict to w>−1/2, ensuring a monotonic behaviour of κ(R) and physically admissible shell stresses (see Section 3).

### 2.2. Apparent-Horizon Condition in Spherical Symmetry

In spherical symmetry, marginally trapped two-spheres are characterized by vanishing outgoing null expansion, which is equivalent to $\nabla_{a}r\;\nabla^{a}r=0$. For the static metrics (1) this condition reduces to $f_{\pm }\left(r\right)=0$, i.e., apparent-horizon radii coincide with the roots of the lapse functions $f_{\pm }$. In this work we restrict the shell evolution to the static patches $f_{\pm }\left(R\right)>0$, so that the shell remains timelike throughout the domain of interest. Consequently, no apparent horizon is contained in the shell-supported region analyzed here; trapping boundaries lie at $f_{\pm }=0$ outside the considered patch. This makes explicit that the present analysis is horizon-avoiding within the chosen static domains, while the location of marginally trapped surfaces is fixed by the roots of $f_{\pm }$.

### 2.3. Balance Scale and Effective Potential Features

An important radius in the evolution is the balance scale

(10) $$R_{\mathrm{thr}}:={\left(\frac{3M}{\Lambda_{-}}\right)}^{1/3},$$

which marks where interior and exterior contributions to the junction become comparable. This expression follows from equating the leading de Sitter term $∼H^{2}R$ in the interior with the Schwarzschild term $∼M/R^{2}$ in the case $\Lambda_{+}=0$ (so that $H^{2}=\Lambda_{-}/3$ and $H^{2}R^{3}≃M$ at the balance point). For $\Lambda_{+}>0$ the precise balance radius is shifted by corrections of order $\Lambda_{+}M^{2}$, but $R_{\mathrm{thr}}$ remains a useful reference scale within the static patch considered here.

A local analysis of the effective potential at this radius shows

(11) $$V^{\prime }\left(R_{\mathrm{thr}}\right)>0\;\Rightarrow \;\ddot{R}=-\frac{1}{2}V^{\prime }\left(R_{\mathrm{thr}}\right)<0\;\left(\dot{R}\neq 0\right),$$

so $R_{\mathrm{thr}}$ acts as a deceleration scale. It does not, however, define a boundary for allowed outward motion.

Outward evolution is only possible where V(R)≤0; the outermost real root $R_{*}$ of V(R)=0 determines the maximal extent of such motion. Thus $R_{\mathrm{thr}}$ indicates the onset of local deceleration, while the full dynamical domain is globally set by the structure of V(R).

## 3. Deceleration Properties at the Balance Scale

Having established the effective potential framework, we now examine the critical radii that govern shell dynamics. The structure of V(R) from Equation (5) reveals a characteristic scale where competing forces reach equilibrium, providing insight into the transition between different evolutionary regimes.

For a shell with surface energy density σ and pressure p=wσ (with w>−1/2), and a fixed exterior mass *M* with a de Sitter interior $\Lambda_{-}>0$, the balance scale defined in Equation (10) marks where interior and exterior contributions are comparable. The restriction w>−1/2 is technically convenient and physically natural: inserting $\sigma \left(R\right)\propto R^{-2(1+w)}$ from Equation (9) into κ(R)=4πσR yields

(12) $$\kappa^{\prime }\left(R\right)=\frac{d}{dR}\left(4\pi \sigma R\right)=4\pi \sigma \left(-1-2w\right),$$

so that for σ>0 and w>−1/2 one has $\kappa^{\prime }\left(R\right)<0$. This ensures a monotonic decrease of κ(R) with radius and simplifies the sign structure of the effective potential without violating standard surface energy conditions for −1≤w≤1.

At the balance radius $R_{\mathrm{thr}}$, the leading interior (de Sitter) and exterior (Schwarzschild) contributions are comparable. Writing the junction dynamics as in Equation (4), the deceleration condition from Equation (11) shows that $R_{\mathrm{thr}}$ is a deceleration scale: near this radius outward motion is slowed down whenever $V^{\prime }\left(R_{\mathrm{thr}}\right)>0$. It does not, however, define a boundary for allowed outward motion. Outward evolution is only possible where V(R)≤0; the outermost real root $R_{*}$ of V(R)=0 determines the maximal extent of such motion. Thus $R_{\mathrm{thr}}$ provides a sufficient local indicator for the onset of deceleration, while the full dynamical domain is globally set by the structure of V(R).

### Thermodynamic Reading

Near $R_{\mathrm{thr}}$ the entropy-like area functional $S_{\mathrm{shell}}=\pi R^{2}$ grows more slowly, reflecting the deceleration of the shell. This motivates $R_{\mathrm{thr}}$ as a dynamically accessible entropy scale in classical collapse configurations, consistent with—but not altering—the purely geometric derivations above.

## 4. Allowable Radial Domain for Outward Evolution

While the balance radius $R_{\mathrm{thr}}$ from Equation (10) provides local insight into shell deceleration, understanding the global evolution requires determining the complete domain where outward motion remains classically permitted. This analysis depends crucially on the full structure of the effective potential and its roots.

The shell’s trajectory is governed by the energy condition in Equation (4), where classical evolution is only permitted in regions satisfying V(R)≤0. The effective potential from Equation (5), derived following standard thin-shell techniques [15], determines this domain through its root structure. In addition, we always restrict to the static patches $f_{\pm }\left(R\right)>0$, so that any radius in the physical evolution domain must lie in a region where the shell remains timelike.

Using the metric functions from Equation (2) and the surface tension combination from Equation (3), the outermost root $R_{*}$ of V(R)=0 in the static patch $f_{\pm }\left(R\right)>0$ bounds the allowable outward domain.

For a linear surface equation of state p=wσ (w>−1/2), the scaling relation from Equation (9) gives $\kappa \left(R\right)\propto R^{-(1+2w)}$. Steeper decay of σ(R) (larger *w*) suppresses κ and tends to widen the domain V(R)≤0, while slower decay contracts it. Initial data with $R\geq R_{*}$ and $\dot{R}\geq 0$ permit outward evolution over finite intervals, possibly with local deceleration effects near $R_{\mathrm{thr}}$.

### Summary

The kinematic condition V(R)≤0 defines the region where classical shell motion is allowed, subject to the static-patch restriction $f_{\pm }\left(R\right)>0$. Deceleration near $R_{\mathrm{thr}}$ affects the rate $\dot{R}$, but does not by itself preclude outward evolution provided the geometric constraint V(R)≤0 continues to hold.

## 5. Boundedness of Curvature Scalars

Having established the dynamical constraints on shell motion, we now address a fundamental question for any classical gravitational model: does the construction remain free of curvature singularities throughout the physically relevant domain? The composite nature of the spacetime—interior, exterior, and junction—requires careful analysis of each component.

In the present thin-shell construction, the spacetime consists of three distinct regions, each admitting direct computation of curvature invariants. In the interior region (de Sitter), the Riemann tensor satisfies

(13) $$R_{\mu \nu \alpha \beta }=\frac{\Lambda_{-}}{3}\left(g_{\mu \alpha }g_{\nu \beta }-g_{\mu \beta }g_{\nu \alpha }\right),$$

which yields a constant Kretschmann scalar

(14) $$K_{\left(\mathrm{dS}\right)}:=R_{\mu \nu \alpha \beta }R^{\mu \nu \alpha \beta }=\frac{8}{3}\;\Lambda_{-}^{2}<\infty .$$

For the exterior Schwarzschild or Schwarzschild–de Sitter region, curvature scalars remain finite for all r≥R(τ) along the shell worldline, provided the shell radius stays bounded away from the central singularity. We therefore define

(15) $$R_{\min}:=\underset{\tau }{\inf}\;R\left(\tau \right)>0$$

over the interval of evolution considered here, i.e., initial data and dynamics are assumed such that the shell never reaches r=0 and remains in the static patches $f_{\pm }\left(R\right)>0$. With this definition, all exterior curvature invariants are finite for $r\geq R\left(\tau \right)\geq R_{\min}>0$, and the potentially singular point r=0 lies outside the shell-supported static domain. In what follows, the regularity statement is explicitly restricted to scalar curvature invariants within the domain covered by the construction, namely the de Sitter interior, the timelike shell worldvolume, and the exterior region with $r\geq R_{\min}>0$ in the static patch $f_{\pm }\left(R\right)>0$, under the assumptions $R\left(\tau \right)\geq R_{\min}>0$ and finite surface stresses.

The shell itself constitutes a distributional surface with well-defined intrinsic and extrinsic geometry. Since evolution is confined to the static patch $f_{+}\left(R\right)>0$, and the Israel junction conditions ensure that discontinuities appear as finite jumps in extrinsic curvature rather than curvature blow-ups, all relevant scalar curvature invariants constructed from the regular parts of the Riemann tensor remain bounded along the shell trajectory for admissible equations of state with finite σ(R) and p(R).

Thus, within the shell-supported static domain and under the assumption $R\left(\tau \right)\geq R_{\min}>0$, the composite spacetime avoids classical curvature singularities in the sense that all scalar curvature invariants stay finite throughout the physical evolution region. We do not analyze global geodesic completeness here; our statements are restricted to curvature boundedness in the domain covered by the timelike thin-shell construction.

## 6. Mass-Scaled Frequency Cutoff

With the shell dynamics and geometric properties established, we now derive an observational consequence that provides a concrete test of the framework. This prediction emerges from combining the classical causal structure near the shell with fundamental limits on information storage and transmission.

The derivation begins with a minimal near-shell storage condition: physical modes localized near the shell cannot be arbitrarily compressed, yielding

(16) $$k_{\mathrm{loc}}\;R≲\xi ,$$

with ξ=O(1). Here $k_{\mathrm{loc}}$ denotes the local proper wavenumber (or spatial momentum scale) of a mode measured by observers comoving with the shell, and *R* is the areal radius. This implies $\omega_{\mathrm{loc}}≃k_{\mathrm{loc}}≲\xi /R$ in natural units.

For static observers at infinity, the Tolman redshift relation connects local and asymptotic frequencies:

(17) $$\omega_{\infty }=\sqrt{f_{+}\left(R\right)}\;\omega_{\mathrm{loc}},$$

where $f_{+}\left(R\right)$ is given by Equation (2). With $f_{c}=\omega_{\infty }/\left(2\pi \right)$ and the Schwarzschild radius $R_{S}=2M$, this yields

(18) $$f_{c}R_{S}\;≲\;\frac{\xi }{2\pi }\;\frac{\sqrt{1-\frac{2M}{R}-\frac{\Lambda_{+}R^{2}}{3}}}{R/R_{S}}.$$

For stationary configurations, Tolman’s law gives $T_{\infty }=\sqrt{f_{+}\left(R\right)}\;T_{\mathrm{loc}}\left(R\right)$.

For vanishing exterior cosmological constant ($\Lambda_{+}=0$), the right-hand side achieves its maximum at $R=\frac{3}{2}R_{S}$, yielding the universal bound

(19) $$f_{c}R_{S}\;\leq \;\frac{\xi }{3\sqrt{3}\;\pi }.$$

Any $\Lambda_{+}>0$ further tightens this constraint within the static patch.

The dimensionless coefficient ξ (denoted by ζ in the original formulation of the universal bound) encodes details of the localization criterion for near-shell modes. Classical general relativity fixes the scaling $f_{c}R_{S}=O\left(1\right)$ but does not determine the precise numerical value of ξ, which is expected to be of order unity. For practical tests one may either adopt a fiducial value, e.g., ξ≃1, or treat ξ as a phenomenological parameter to be constrained by observations of persistent near-shell spectral features.

### Falsifiability

Given an independent mass determination, any persistent near-shell spectral feature observed at infinity must respect the bound (19) within the chosen range of ξ. A robust violation ($f_{c}R_{S}\gg \xi /\left(3\sqrt{3}\;\pi \right)$) would falsify the classical near-shell storage picture under the stated assumptions (timelike shell, static patches, negligible inflow, time-independent exterior mass).

## 7. Robustness to Small Inflow

Finally, we examine the stability of our predictions under more realistic conditions where the idealized assumption of strictly constant exterior mass is relaxed. This analysis addresses whether the frequency bound and other results remain meaningful in the presence of weak, adiabatic perturbations.

Consider a slowly varying exterior mass M(τ) and shell radius R(τ). The instantaneous outward domain remains defined by V(R)≤0 on the static patch, with M(τ) now entering the metric function $f_{+}\left(R\right)$ from Equation (2).

### 7.1. Adiabatic Robustness

For slowly varying system parameters (adiabatic regime),

(20) $$\left|\frac{\dot{M}}{M}\right|\;\ll \;2\pi f_{c}\;,\;\left|\frac{\dot{R}}{R}\right|\;\ll \;2\pi f_{c}\;,$$

the instantaneous bound from Equation (19) remains predictive when $R_{S}\left(\tau \right)=2M\left(\tau \right)$. Outside this regime, nonstationarity smears persistent features; one should test time-window averages or envelopes of $f_{c}R_{S}\left(\tau \right)$.

### 7.2. Balance Functional

To quantify the robustness more precisely, define $F\left(\tau \right)=H^{2}R^{3}-M\left(\tau \right)$ with $H^{2}=\Lambda_{-}/3$. Its evolution,

(21) $$\dot{F}=\frac{R^{3}}{3}\;{\dot{\Lambda }}_{-}+3H^{2}R^{2}\;\dot{R}-\dot{M}\;,$$

remains positive whenever $\dot{M}<{\dot{M}}_{\mathrm{crit}}=\frac{R^{3}}{3}{\dot{\Lambda }}_{-}+3H^{2}R^{2}\dot{R}$, preserving F≥0 locally in time and favoring outward evolution provided V(R)≤0 holds.

## 8. Discussion and Outlook

This study has analyzed late-time gravitational collapse in classical general relativity using a spherically symmetric timelike thin-shell construction, with dynamics governed entirely by junction conditions and classical geometry. The framework reveals that classical general relativity naturally supports thermodynamic-like behavior during collapse, offering insights into gravitational coarse-graining without invoking quantum corrections or exotic matter.

### 8.1. Thermodynamic Framework and Classical Entropy Production

The thermodynamic interpretation used in this work is deliberately macroscopic and classical. It is based on two geometric ingredients that are already present in the junction description: (i) the area scaling of the shell, $A=4\pi R^{2}$, and (ii) Tolman redshift, which relates local quantities measured by observers comoving with the shell to those seen at infinity. We emphasize that the resulting entropy-like functional introduced below is a coarse-grained, geometric bookkeeping quantity; it is not a microstate-counting entropy derived from an underlying statistical ensemble.

Starting from the exact surface energy balance (7), and using

(22) $$\frac{dA}{d\tau }=8\pi R\dot{R},$$

one may introduce an entropy-like area functional

(23) $$S_{\mathrm{shell}}:=\alpha R^{2}\;\left(\alpha >0\right),$$

together with a local temperature $T_{\mathrm{loc}}\left(R\right)$ defined at the shell and related to asymptotic measures by Tolman’s law (Section 6). With these definitions, (7) can be rearranged into the first-law–type form

(24) $$\frac{dS_{\mathrm{shell}}}{d\tau }=\frac{A\;\Phi }{T_{\mathrm{loc}}\left(R\right)}+2\alpha R\;\dot{R},\;A=4\pi R^{2},$$

where the term $A\Phi /T_{\mathrm{loc}}$ represents the flux contribution (with Φ as defined in Equation (8)) and the term $2\alpha R\dot{R}$ is the purely geometric area-growth contribution. Equation (24) is thus a rearrangement of (7); its content is fixed by the junction conservation law once $S_{\mathrm{shell}}\propto R^{2}$ is adopted as the entropy-like coarse-grained variable.

In the post-transient regime of negligible net inflow (Φ≃0), Equation (24) reduces to

(25) $$\frac{dS_{\mathrm{shell}}}{d\tau }=2\alpha R\dot{R},$$

so outward evolution ($\dot{R}>0$) corresponds to monotonic growth of the entropy-like area functional within the static patches considered here. This provides a controlled classical analogy to area-based thermodynamic descriptions, without invoking event-horizon formation or microscopic state counting.

### 8.2. Coarse-Graining and Gravitational Entropy

The entropy-like functional $S_{\mathrm{shell}}\propto R^{2}$ should be understood as a coarse-grained measure of gravitational disorder, not as a fundamental statistical entropy obtained from microstate counting. Coarse-graining is implemented at the classical level by averaging over small fluctuations in the shell’s position and stress tensor, in the spirit of quasiclassical histories and effective descriptions of gravitational entropy [6,7,8]. In this sense, the thin-shell model provides a classical analogue of area-type entropy relations: the relevant coarse-grained quantity scales with an area, while the underlying microscopic degrees of freedom remain unspecified.

This perspective is complementary to more microscopic or quantum-motivated frameworks, such as loop-quantum-gravity calculations of black hole entropy or entanglement entropy of quantum fields across spatial regions [6,8]. In this sense, the present construction offers a simple classical setting in which an area-based entropy-like functional emerges naturally from the junction geometry and surface conservation laws, and it delineates what can already be said within classical general relativity at a macroscopic, coarse-grained level, complementary to more microscopic, quantum-based treatments.

### 8.3. Comparison to Black Hole Thermodynamics

The classical entropy-like production encoded in (24) bears qualitative similarities to black hole thermodynamics, yet operates in a distinct, horizon-avoiding regime. In the semiclassical picture, black hole entropy is tied to horizon area and Hawking radiation, and the area theorem constrains the evolution of event horizons [1,2,17]. In the present model, no event horizon is formed in the shell-supported static domain, and curvature invariants remain bounded provided $R\left(\tau \right)\geq R_{\min}>0$ and $f_{\pm }\left(R\right)>0$ hold throughout the evolution.

Nevertheless, the Tolman redshift factor $\sqrt{f_{+}\left(R\right)}$ and the area-scaling of $S_{\mathrm{shell}}$ mirror familiar features from horizon thermodynamics: local temperatures increase as *R* approaches $R_{S}$, and area growth along outward evolution resembles classical area theorems. The key difference is that these effects arise here from a timelike shell in a regular, static patch rather than from a null event horizon, so the comparison is explicitly restricted to classical analogies and does not address quantum particle creation or detailed semiclassical black hole thermodynamics.

### 8.4. Transport Properties and Membrane Paradigm

The entropy production framework (24) naturally connects to membrane paradigm treatments of horizon physics, in which horizons are modeled as timelike surfaces with effective transport properties [18]. In the present context, the timelike shell can be viewed as a classical membrane with effective transport coefficients determined by the surface stress tensor S  ba=diag(−σ,p,p) and the junction conditions.

One may define effective surface transport coefficients such as a surface viscosity, bulk surface viscosity, and an effective conductivity (schematically),

(26) $$\begin{array}{cc} \eta_{s} & ∼\sigma R^{2}, \end{array}$$

(27) $$\begin{array}{cc} \zeta_{s} & ∼\left(\sigma +2p\right)R^{2}, \end{array}$$

(28) $$\begin{array}{cc} \sigma_{\mathrm{cond}} & ∼\frac{R^{2}}{\kappa (R)}, \end{array}$$

which parametrize dissipative corrections to the shell evolution and connect to the flux term Φ in Equation (7) through constitutive relations. These quantities should be understood as effective, coarse-grained parameters describing the macroscopic response of the shell, rather than as transport coefficients derived from an underlying microstate model. Unlike traditional membrane treatments that focus on null horizons, this classical approach operates entirely in the static patch and admits full analytical control.

### 8.5. Information-Theoretic Implications

The bounded curvature and absence of event horizons or central singularities within the shell-supported static domain have implications for how information can be stored and transported in this model. The maximum value of the entropy-like functional $S_{\mathrm{shell}}\propto R^{2}$ along a given evolution can be interpreted as a bound on the amount of coarse-grained information that can be associated with the shell configuration, in analogy with area-based information capacity bounds [1].

The mass-scaled frequency bound $f_{c}R_{S}\leq \xi /\left(3\sqrt{3}\pi \right)$ derived in Section 6 adds a complementary constraint: long-lived, near-shell modes observed at infinity cannot exceed this dimensionless combination within the stated assumptions. Modes that significantly violate the bound would lie outside the classical near-shell storage picture developed here. In this work, information-theoretic statements are restricted to classical fields and coarse-grained observables; issues such as quantum information, unitarity, or fine-grained entropy are left for future extensions.

### 8.6. Observational Implications and Falsifiability

The frequency bound offers a direct observational handle: persistent spectral features from ultracompact objects should respect

(29) $$f_{c}R_{S}\;≲\;\frac{\xi }{3\sqrt{3}\pi },$$

for some ξ=O(1) determined by the localization criterion for near-shell modes. Given an independent estimate of the mass *M*, any systematic violation of this inequality by long-lived features would falsify the classical near-shell storage scenario under the assumptions used in the derivation (timelike shell, static patches, negligible inflow, time-independent exterior mass).

Current and future gravitational-wave and electromagnetic observations provide natural arenas for testing such bounds, for instance through late-time quasi-periodic signals, ringdown tails, or echo-like features associated with ultracompact objects [19,20]. While the present model is highly idealized, it illustrates how classical junction geometries can lead to concrete, mass-scaled observables that can, in principle, be confronted with data.

### 8.7. Future Directions

The current framework provides a fully analytical, purely classical description of collapse in a highly symmetric setting. Many aspects of real astrophysical systems, such as asymmetries, evolving interior matter, or finite-thickness shells, go beyond this idealisation. Addressing these issues would require numerical methods or new analytical techniques, but the structure developed here could serve as a stable foundation for such extensions. From a theoretical perspective, exploring analogies with membrane-based descriptions of black hole dynamics could be fruitful, as these studies examine classical transport properties and entropy production without invoking quantum gravity. Investigating whether similar mechanisms apply to timelike shells could enhance the thermodynamic interpretation and facilitate the connection of geometric collapse models to broader frameworks in relativistic field theory. Future work could also incorporate quantum fluctuations or additional matter degrees of freedom, thereby aligning more closely with holographic or string-inspired models when appropriate.

At the same time, the present analysis makes explicit that the entropy-like functional $S_{\mathrm{shell}}\propto R^{2}$ is purely coarse-grained and geometric: it does not resolve any underlying microstates or provide a microscopic state counting. In this sense, the thin-shell model delineates the boundary of what can be said within classical general relativity alone. Any attempt to go beyond this level and attribute a detailed microstructure to $S_{\mathrm{shell}}$ must introduce additional ingredients—such as quantum fields, modified geometries, or new degrees of freedom—so that the present framework can serve as a classical benchmark for more microscopic proposals.

## Data Availability

All derivations and analytical results in this work follow directly from the equations presented in the manuscript. No external datasets were used or generated.
